# Cancer-directed surgery brings survival benefits for patients with advanced prostate cancer: a population-based propensity-score matching study

**DOI:** 10.7150/jca.80232

**Published:** 2023-01-01

**Authors:** Ru Chen, Yan Lu, Shigeo Horie, Monica Vogel, Ruochen Zhang, Ping Zheng, Yongbao Wei

**Affiliations:** 1 Department of Urology, The First Hospital of Putian City, Putian, Fujian, China.; 2 Department of Urology, Juntendo University School of Medicine, Tokyo, Japan.; 3 University of Chicago, Chicago, Illinois, USA.; 4 Shengli Clinical Medical College of Fujian Medical University, Fuzhou, 350001, China.; 5 Department of urology, Fujian Provincial Hospital, Fuzhou, 350001, Fujian, China.; 6 Department of Urology, Shangrao municipal Hospital, Shangrao, Jiangxi Province, China.

**Keywords:** Survival benefit, cancer mortality, cancer-directed surgery, prostate cancer, population-based study

## Abstract

**Purpose:** The purpose of this study was to evaluate the survival benefits of cancer-directed surgery (CDS) for localized prostate cancer (PCa) as well as advanced PCa.

**Methods:** We retrospectively used the Surveillance, Epidemiology, and End Results (SEER) database and conducted a propensity score matching (PSM) study to investigate survival benefits and influencing factors of CDS in patients with PCa, especially for those with advanced PCa.

**Results:** 19,729 cases were included. Patients who were recommended CDS had lower stages of disease (81.01% vs. 77.32% at stages I and II, p<0.01) than those who were not recommended CDS. It was primarily age, diagnosis year, cancer stage (American Joint Committee on Cancer Staging System), Gleason score, race, and home location and prostate-specific antigen, that influenced whether CDS was recommended or not (all p<0.05). Patients with PCa had lower rates of cancer specific mortality (CSM) and overall mortality (OM) when CDS was performed (CDS performed=CDSP). The unselected patients with CDSP decreased both rates of CSM by 79% and OM by 26% (both p<0.001). CDSP also benefited the young patients (with age ≤74 years old) with stage IV disease, promoting a rate decrease by 28% in CSM and by 31% in OM (both p<0.001).

**Conclusions:** We found a decline in CSM and OM for unselected patients with PCa and patients less than 74 years old with stage IV disease. CDS as part of a multimodal treatment concept should be considered for an alternative treatment for patients with advanced PCa.

## Introduction

Cancer-directed surgery (CDS) is an important treatment for solid cancers, and it appears to provide both survival and therapeutic benefits 1,2. Several cancer-directed surgeries, such as radical prostatectomy (RP), cytotherapeutic ablation, focal photodynamic therapy, and surgical treatment for metastases, play an important role in the management of prostate cancer (PCa) 3. However, while CDS provides potential benefits to patients with many kinds of solid tumors, there were a considerable number of patients who were not recommended to undergo CDS, or who refused the treatment for varying reasons 2,4,5. CDS such as RP is considered a curative treatment for localized PCa, while it may also be used simply as one part of a multimodal therapeutic regimen for selected patients with local advanced PC and limited benefits for likelihood of survival. However, the potential benefit of CDS for metastatic PCa remains contradicttory 3,6,7. Therefore, it is of great clinical significance to analyze the role and efficacy of CDS in treating PCa patients. In this study, we investigated the potential benefits and influencing factors of CDS in PCa patients, particularly those with advanced stages of the disease.

## Methods

### Study design and patients

The Surveillance, Epidemiology, and End Results (SEER) data records of CDS in cancer patients are useful for many types of cancer, including prostate 8. CDS includes curative surgeries for primary solid cancers (such as RP), but also focal therapy (such as metastasectomy) for metastatic disease 9. We downloaded the PCa database in June 2022. The site record of prostate and cancer stage of American Joint Committee on Cancer Staging System (AJCC) (7th edition) were searched and included. The PCa patients who met the following inclusion criteria were included: (a) patient age above 40 years, and (b) their diagnosis year between 2010 and 2019. The patients who met one of the following exclusion criteria were excluded from the study: (a) died before surgery recommended; or (b) with survival months less than three months; or (c) with unknown status of survival. We set up two case-control cohorts to study the reasons why CDS was recommended (CDSR) or not (CDSnR). CDSR was defined as the following items: surgery performed, surgery unknown if performed or recommended but not performed due to unknown reason, and recommended but not performed due to patient refusal. CDSnR was defined as patients who were not recommended to undergo CDS by medical service providers, regardless of whether the patients did or did not undergo the surgery, representing the willingness of service providers to recommend CDS. We also excluded certain data from the scope of the study: cases with an unknown death certificate, autopsy only, or those who died before recommended surgery; and survival time of fewer than three months. Furthermore, we also set up another two case-control cohorts to study the reasons and benefits for those who underwent CDS (CDS performed = CDSP) or did not (CDS not performed =CDSnP). The diagnosis year was divided into two groups according to their medium: 2010-2015 and 2016-2019. The American Joint Committee on Cancer (AJCC) stage, mainly based on TNM staging, was used for cancer stages, with scores ranging from 1-4 10. The Gleason score (GS) was bioptic and was divided into three groups: ≤6, =7, and 8-10. The prostate-specific antigen (PSA) was divided into the following three groups: 98.00 ng/ml or greater; 0.10 or fewer nanograms/milliliter (ng/ml), and others (PSA tested but results not presented in the data). Race was divided into the following three groups: white, black, and others. Income was divided into three groups according to median: <$70,000, ≥$70,000, and missing value. Patient home locations were grouped according to the counties' populations: big city (more than 1 million population), small city (less than 1 million population), and missing value. Cancer-specific mortality (CSM) was derived from the "SEER cause-specific death classification", where the rate of deaths caused by the cancer itself was defined as prostate cancer-specific mortality. Overall mortality (OM) was defined according to the visual status of survival and divided into two opposite outcomes: dead and alive.

### Statistical analysis and methods

We used IBM SPSS Statistics 27.0 for the data analyzed. Comparisons between two groups were made using the two independent samples test of the nonparametric test before and after propensity score matching (PSM). We made comparisons between the two cohort groups (CDSR vs. CDSnR; CDSP vs. CDSnP) with a 1:1 matching ratio. We used the following several items for matching: age, diagnosis year, race, income, home location, GS, and AJCC stage. PSA and clinical TNM stage were not used for matching as their missing records were more than 50%. Following this, the influencing factors were analyzed by binary logistic regression analysis. The analysis of OM and CSM and their plots were performed using Kaplan-Meier alongside Log Rank by R-language (Version 4.2.1). We then performed a multivariable Cox proportional hazard model for CSM and OM for CDSP by two models, the adjusted model 1 was used to adjust for age, AJCC stage, GS, and race. The adjusted model 2 was used to adjust for age, AJCC stage, GS, race, diagnosis year, income, and home location. A p-value less than 0.05 was considered statistically significant.

## Results

### Demographic data

**Fig 1:**
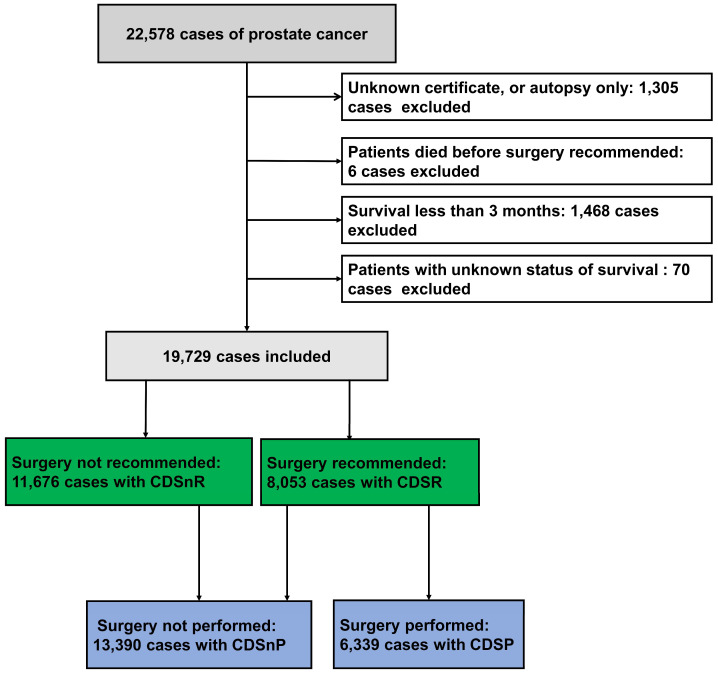
Patients with prostate cancer from the SEER database. 11,676 patients had CDSnR, and 8,053 patients had CDSR. 13,390 patients had CDSnP, and 6,339 patients had CDSP. Abbreviations: CDSR= cancer-directed surgery recommended; CDSnR= cancer-directed surgery not recommended; CDSP= cancer-directed surgery performed; CDSnP= cancer-directed surgery not performed. SEER= the Surveillance, Epidemiology, and End Results

A total of 22,578 PCa cases diagnosed between 2010 and 2019 were analyzed in this study. After considering inclusion and exclusion criteria, 19,729 cases were finally included (Fig. 1), which consisted of 8,053 CDSR cases (40.82%) and 11,676 CDSnR cases (59.18%). There were significant differences in patient age, diagnosis year, clinical T stage, clinical M stage, GS, PSA, AJCC stage, race, income, home location, and survival time when comparing the two groups (CDSR and CDSnR) before PSM (Table 1, all p<0.05). After PSM by 1:1 ratio, we included 3,581 cases for each group. The comparison results indicated significant differences between patients with CDSR and those with CDSnR with regards to GS (p<0.01), as well as AJCC stage, PSA, CSM, OM, and survival time (all p<0.001); however, no significant differences was observed in age, diagnosis year, race, income, and home location (all p>0.05). Compared to patients with CDSnR, patients with CDSR had, lower AJCC stage (81.01% vs. 77.32% at stages I and II, p<0.001), lower rates of CSM (10.56% vs. 17.96%) and OM (27.87% vs. 33.01%), and longer medium survival time, 76.00 months (m.) vs. 71.00 m. (all p<0.001), with IQR of 52.00 m. to 100.00 m. and IQR of 50.00 m. to 95.00 m., respectively.

### Influencing factors

We analyzed age, home location, race, income, AJCC stage, GS, and PSA as independent variables as influential factors on whether CDS was recommended or not for unselected patients after PSM. We found that age, GS, and PSA were negative impact on CDSR, while diagnosis year, race, AJCC stage, and home location were positive influential factors (Table 2, all p<0.05) (PSM data can be accessed in supplementary data 1).

We then analyzed the factors influencing CDSR or CDSnR in young patients (≤74y.) with AJCC stage IV PCa. The patients we included in this part met the following criteria: (1) aged 74 years and younger, and (2) AJCC stage IV (7th edition). The AJCC stage IV disease included T4 N0 M0, N1, and M1 with any PSA or any GS according to its definition 10. The total number of eligible patients in the two groups was 1,211, with 231 (19.01%) in the CDSR group and 980 (80.92%) in the CDSnR group. There were significant differences in home location, GS, PSA, CSM, OM, and survival time (all p<0.05), whereas there were no significant differences in age, diagnosis year, income, and race between the two groups. The total number of cases after PSM was 270; 135 cases each for CDSR and CDSnR. We only found PSA (98.00 ng/ml or greater) was negative significant influential factors on CDSR (p<0.05) (Table 3).

### Survival Comparisons

**Fig 2:**
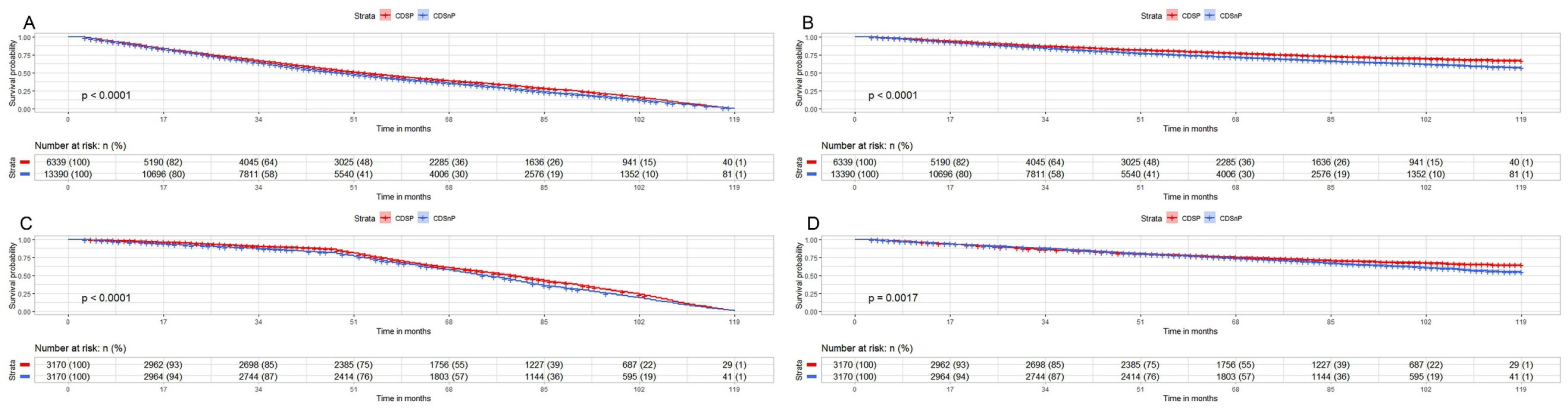
Impact of CSDP or CDSnP in unselected PCa patients from SEER data base with PCa diagnosis from 2010 to 2019 on survival. Shown are Kaplan-Meier curves before (patients with CDSP n=6,339; patients with CDSnP n=13,390) and after PSM (patients with CDSP n=3,170; patients with CDSnP n=3,170). A: CSM of unselected patients before PSM. B: OM of unselected patients before PSM. C: CSM for unselected patients after PSM. D: OM of unselected patients after PSM (All p<0.001). Abbreviations: PCa=prostate cancer; CSM=cancer-specific survival; OM=overall survival; PSM= propensity score matching by 1:1 ratio; time=months; CDSP= cancer-directed surgery performed; CDSnP= cancer-directed surgery not performed.

We analyzed the CMS and OM for unselected patients (CDSnP patients n=13,390; CDSP patients n=6,339) by Kaplan-Meier analysis. We found significantly lower rates of CSM (7.17% vs. 13.69%) and OM (20.82% vs. 23.60%) (both p<0.001) in the CDSP group after 120 months, compared with the group of CDSnP (Fig. 2A and 2B). The patients in the CDSP group decreased 79% and 26% in both CSM and OM (both p<0.001), respectively, compared to those in the CDSnP group, according to adjusted model 2 before PSM. After PSM, we obtained a total of 6340 cases (3170 cases with CDSP and 3170 cases with CDSnP) to analyze OM and CSM (The PSM data can be accessed in supplementary data 2). The rates of CSM (10.56% vs. 17.96%) and OM (27.87% vs. 33.01%) were lower in patients with CDSP than those with CDSnP after 120 months (both p<0.001) (Fig. 2C and 2D). However, the patients with CDSP decreased by about 28% and 31% in CSM and OM according to PSM adjusted model 2 (both p<0.001) (Table 4).

**Fig 3:**
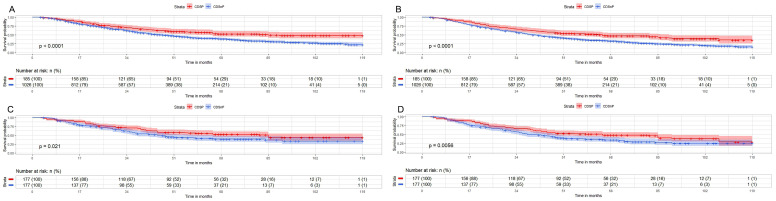
Impact of CSDP or CDSnP in young PCa patients (≤74 years old) with AJCC stage IV disease from SEER data base with PCa diagnosis from 2010 to 2019 on survival. Shown are Kaplan-Meier curves before (patients with CDSP n=185; CDSnP n=1,026) and after PSM (patients with CDSP n=177; patients with CDSnP n=177). A: CSM of these PCa patients before PSM. B: OM of these PCa patients before PSM. C: CSM for these PCa patients after PSM. D: OM of these PCa patients after PSM (All p<0.05). Abbreviations: CSM=cancer-specific survival; OM=overall survival; PSM= propensity score matching by 1:1 ratio; time=months; CDSP= cancer-directed surgery performed; CDSnP= cancer-directed surgery not performed.

We then analyzed the survival outcomes of CDSP or CDSnP for young patients (≤74y.) with AJCC stage IV PCa. A total of 1,211 cases (185 cases with CDSP and 1,026 cases with CDSnP) were analyzed for CSM (Fig. 3A) and OM (Fig. 3B). The rates of CSM (44.86% vs. 60.14%) and OM (54.05% vs. 71.15%) were lower in patients with CDSP than those with CDSnP after 120 months before PSM (both p<0.05). A total of 354 cases (177 cases with CDSP and 177 cases with CDSnP) were included after PSM to analyze OM and CSM (The PSM data can be accessed in supplementary data 3). The rates of CSM (40.02% vs. 55.93%) and OM (55.93% vs. 66.67%) were lower in patients with CDSP than those with CDSnP after 120 months (both p<0.01) (Fig. 3C and 3D). The patients with CDSP decreased by about 26% and 31% mortality risk in CSM and OM (both p<0.05), respectively, according to PSM adjusted model 2 (Table 5).

## Discussion

There are many reasons why a patient may not be recommended to pursue CDS as a treatment for their cancer, or why a patient may refuse the treatment. One study found that in patients with colon cancer, refusal of CDS was closely related to patient race, gender, and insurance status 2. Another study that included non-metastatic ten common solid cancers, including PCa, also found that insurance and marital status played a key role in racial and ethnic differences in the refusal of CDS 11. In this study, we did not include insurance status or marital status as concerns any more. Similar to previous studies 2,11, we found that there was a great difference between patients with CDSR and patients with CDSnR for different stages of PCa. For unselected PCa patients, whether CDSR or not was closely related to age, stage, race, income, and home location. Among them, age, GS, and PSA were found as negative impact on CDSR, while the rest of them were considered as positive influential factors. Patients with CDSR had a median age of 68.00 y., as curative intended therapy is only recommended from a statistical life expectancy of at least ten years 12. Interestingly, we found that for young patients (with age ≤74y.) with AJCC stage IV disease, only PSA (98.00 ng/ml or greater) was negative significant influential factors on CDSR, which usually indicated a poor outcome 3. Whether CDSR or not to these patients is a challenge to the existing urological guidelines 3,7. Thus, medical service providers need to carefully select suitable patients for rigorous plans of treatment and follow-up during daily clinical practice.

Screening and positive treatment of clinically significant PCa are important for the management of PCa, as undifferentiated PSA screening reduces PCa-related mortality but has little effect on all-cause mortality 13. Therefore, for decades, non-CDS treatment modalities but active surveillance were recommended for low- and intermediate-risk PCa patients 14. We included patients with advanced PCa, who were referred to clinically significant PCa 15,16. We selected young patients (≤74y.) with AJCC stage IV disease for further analysis, which may be a kind of patient with clinically significant PCa and have to be given positive treatment recommendations 7,12. As for patients with localized PCa, radical radiation therapy (RT) or RP for the primary tumor is a curative treatment and is currently recommended by guidelines if they have a long-life expectancy 12. A large study with a 20-year follow-up confirmed that RP brought survival benefits including metastasis-free survival (MFS) and cancer-specific survival (CSS) for patients with favorable PCa characteristics such as localized PCa with fewer than three positive lymph nodes 17. Men with localized PCa and longer life expectancy benefit from RP, resulting in an average of 2.9 years longer survival time 18. Two reviews further confirmed that RP or RT as a local treatment plus androgen deprivation therapy brought more survival benefits for patients with high-risk localized PCa 19 or clinically positive lymph nodes 20. However, our study found that the proportion of CDSR was about 19%, compared to CDSnR of about 81% in young patients with AJCC stage IV. This proportion seems relatively small, as a considerable number of patients may choose other treatments such as RT and androgen-deprivation therapy (ADT) together 21, or other systemic therapies (taxane-based chemotherapy, etc.) 22. In the present study, our findings (though from limited data) indicated that CDSP produced benefits in CSS and OS for patients with T4 or N1 stage disease, while we observed decreasing mortality risks by 28% in CSM and by 31% in OM (both p<0.05).

Aggressive treatment is more important for severe lethal PCa, especially in patients with M1 PCa, in which CDS including RP is only one part of a multimodal treatment plan 3,23,24. CDS for cancers in SEER data includes the surgical treatment of the primary tumor and the surgical treatment of the metastases 9. In M1 patients, CDS is not usually recommended as a treatment option 3,7. For this kind of patient with primary or metastasis-directed therapy, other options include radiotherapy, radiofrequency ablation, and others. The proportion of patients who accepted surgery was very small 25-27. For M1 patients, CDSP may control symptoms and relieve PSA, but the benefits for actual survival were very limited, with only some patients experiencing those benefits 23,24,28. Our study evaluated OM and CSM benefits in patients with CDSP not limited to primary tumor resection, even though the number and location of metastases, as well as the patient's performance status, were not analyzed. Our limited data confirmed that patients with CDSP had a risk decline in both CSM and OM in young patients with the AJCC IV stage including M1 disease, indicating that CDS may be a potential treatment option for these patients.

Apart from some limitations of the retrospective study, this study did not provide a detailed type of CDS and its impact on survival benefits. We included data with a high missing rate (over 50%) in PSA and TNM staging records, resulting in a possible bias in analysis and related results. We only analyzed the role of CDS and did not study the significance of radiotherapy or combination therapies with or without ADT, even though CDS or RT combined with systemic therapy are more preferred treatments, especially for patients with advanced disease. Furthermore, we only included data from SEER, which only covers part of the population from North America. Thus, we did not explore the role and significance of CDS in PCa patients in other geographic locations, which may yield different trends and results.

Consequently, we analyzed the influence and survival benefits of CDS in the management of patients with PCa. We found a decline in risks for CSM and OM for unselected patients with PCa as well as those less than 74 years old and staged AJCC IV (7^th^ edition). Our study found evidence that CDS as part of a multimodal treatment plan may be a viable alternative treatment for patients with locally advanced or even distant stages of the disease.

## Figures and Tables

**Table 1:** Baseline comparisons between the patients with cancer-directed surgery recommended (CDSR) and not recommended (CDSnR) from the SEER database.

			Before PSM				After PSM	
Variables		CDSR(n=8053)	CDSnR(n=11,676)	P value#		CDSR(n=3581)	CDSnR (n=3581)	P value#
Age (y.) median (IQR)		68.00 (62.00-76.00)	68.00 (62.00-74.00)	p<0.001		68.00 (62.00-75.00)	68.00 (62.00-75.00)	p=0.22
mean±SD		68.78±9.94	68.16±9.13			68.39±9.78	68.22±9.37	
Diagnosis year (y.) N (%)				p<0.001				p=0.96
2010-2015		5066 (62.91)	6506 (55.72)			3581 (100.0)	3581 (100.0)	
2016-2019		2987 (37.09)	5170 (44.28)			0	0	
Race N (%)				p<0.001				p=0.55
White		6662 (82.73)	8383 (71.80)			3053 (85.26)	2986 (83.38)	
Black		675 (8.38)	1871 (16.02)			316 (8.82)	403 (11.25)	
Others		480 (5.96)	800 (6.85)			212 (5.92)	192 (5.36)	
Missing value		236 (2.93)	622 (5.33)			0	0	
Income N (%)				p<0.001				p=1.00
<$70,000		4893 (60.76)	6870 (58.84)			2121 (59.23)	2133 (59.56)	
≥$70,000		3160 (39.24)	4806 (41.16)			1460 (40.77)	1448 (40.44)	
Missing value		0	0			0		
Home location N (%)				p<0.001				p=0.91
Big city		3093 (38.41)	7403 (63.40)			1726 (48.20)	1678 (46.86)	
Small city		4947 (61.43)	4272 (36.59)			1855 (51.80)	1903 (53.14)	
Missing value		13 (0.16)	1 (0.00)					
PSA (ng/ml) N (%)				p<0.001				p<0.001
PSA (others)		1574 (19.55)	3034 (25.98)			701 (19.58)	941 (26.28)	
PSA (98.0 ng/ml or greater)		708 (8.79)	3213 (27.52)			335 (9.35)	887 (24.77)	
PSA (0.1 or less)		51 (0.63)	15 (0.13)			22 (0.61)	4 (0.11)	
Missing value		5720 (70.03)	5414 (46.37)			2523 (70.46)	1749 (48.84)	
Clinical T stage N (%)				p<0.001				-
T1		596 (7.07)	744 (6.37)			0	0	
T2		594 (7.38)	1056 (9.04)			0	0	
T3		208 (2.58)	232 (1.99)			0	0	
T4		36 (0.45)	235 (2.01)			0	0	
Missing value		6615 (82.14)	9409 (80.58)			3581 (100.0)	3581 (100.0)	
Clinical N stage N (%)				p<0.05				-
Nx		252 (3.13)	444 (3.78)			0	0	
N0		1099 (13.65)	1542 (13.21)			0	0	
N1		83 (1.03)	285 (2.44)			0	0	
Missing value		6615 (82.14)	9405 (80.55)			3581 (100.0)	3581 (100.0)	
Clinical M stage N (%)				p<0.001				-
M0		1324 (16.44)	1709 (14.64)			0	0	
M1		110 (1.37)	562 (4.81)			0	0	
Missing value		6615 (82.14)	9405 (80.55)			3581 (100.0)	3581 (100.0)	
Bioptic GS N (%)				p<0.001				p<0.01
≤6		3472 (43.11)	4434 (37.98)			1474 (41.16)	1518 (42.39)	
=7		2730 (33.90)	2998 (25.68)			1185 (33.09)	995 (27.79)	
8-10		1851 (22.99)	4244 (36.34)			922 (25.75)	1068 (29.82)	
AJCC stage N (%)				p<0.001				p<0.001
I		1513 (18.79)	2224 (19.05)			1138 (31.78)	1479 (41.30)	
II		2250 (27.94)	2086 (17.87)			1763 (49.23)	1290 (36.02)	
III		404 (5.02)	78 (0.67)			322 (8.99)	47 (1.31)	
IV		381 (4.73)	1461 (12.51)			358 (10.00)	765 (23.36)	
Missing value		3505 (43.52)	5827 (49.91)			0		
CSM N (%)				p<0.001				p<0.001
Dead		577 (7.17)	1598 (13.69)			378 (10.56)	643 (17.96)	
Alive		7476 (92.83)	10078 (86.31)			3203 (89.44)	2938 (82.04)	
OM N (%)				p<0.001				p<0.001
Dead		1677 (20.82)	2755 (23.60)			998 (27.87)	1182 (33.01)	
Alive		6376 (79.18)	8921 (76.40)			2583 (72.13)	2399 (66.99)	
Survival time (m.) median (IQR)		49.00 (23.00-87.00)	39.00 (20.00-73.00)	p<0.001		76.00 (52.00-100.00)	71.00 (50.00-95.00)	p<0.001

Abbreviations: CDSR= Cancer-directed surgery recommended; CDSnR= Cancer-directed surgery not recommended; GS= Gleason Score; PSA= Prostate-specific antigen; AJCC stage =AJCC Stage 7th edition; PSM= propensity score matching, y.=years old; m.=months; CSM=cancer-specific survival; OM=overall survival; IQR= interquartile range; SEER= the Surveillance, Epidemiology, and End Results. (#) Mann-Whitney Test.

**Table 2:** Influencing factors of cancer-directed surgery recommended (n=8,053) or not (n=11,676) for unselected patients with prostate cancer by binary logistic regression after PSM.

Variables	Regression coefficient	Standard error	Chi-square value	Degrees of freedom	P value	OR	95% CI for OR	
							Lower	Upper
Age	-0.01	0.01	8.37	1	p<0.01	0.99	0.98	0.99
Diagnosis year			15.39	5	p<0.01			
Diagnosis year (1)	0.33	0.15	4.83	1	p<0.05	1.39	1.04	1.87
Diagnosis year (2)	0.48	0.15	10.77	1	p<0.01	1.61	1.21	2.15
Diagnosis year (3)	0.11	0.15	0.51	1	p=0.47	1.12	0.83	1.51
Diagnosis year (4)	0.29	0.15	3.85	1	p=0.05	1.344	1.00	1.81
Diagnosis year (5)	0.42	0.15	7.99	1	p<0.01	1.52	1.14	2.03
Race			9.51	2	p<0.01			
Race (1)	0.07	0.14	0.21	1	p=0.65	1.07	0.81	1.411
Race (2)	0.49	0.16	9.47	1	p<0.01	1.64	1.19	2.24
Income (1)	0.01	0.10	0.01	1	p=0.98	1.00	0.82	1.23
Home location (1)	0.21	0.11	4.12	1	p<0.05	1.24	1.01	1.52
AJCC stage			233.43	3	p<0.001			
AJCC stage (1)	3.13	0.26	148.57	1	p<0.001	22.87	13.83	37.82
AJCC stage (2)	4.78	0.33	207.37	1	p<0.001	119.23	62.20	228.56
AJCC stage (3)	2.72	0.29	89.48	1	p<0.001	15.23	8.66	26.77
GS			60.77	2	p<0.001			
GS (1)	-1.93	0.25	59.95	1	p<0.001	0.145	0.09	0.24
GS (2)	-1.65	0.26	41.71	1	p<0.001	0.19	0.12	0.32
PSA			72.44	2	p<0.001			
PSA (1)	-0.97	0.13	54.59	1	p<0.001	0.38	0.29	0.49
PSA (2)	2.37	0.58	16.75	1	p<0.001	10.64	3.43	33.03
Constant	-0.79	0.34	5.36	1	0.021	0.45		

Abbreviations: GS= Gleason Score; PSA= Prostate-specific antigen; AJCC stage =AJCC Stage 7th edition; CI=confidence interval; OR=odds ratio; PSM= propensity score matching.

**Table 3:** Influencing factors of cancer-directed surgery recommended (n=185) or not (n=1026) for young patients (≤74 years old) with AJCC stage IV prostate cancer after PSM by binary logistic regression.

Variables	Regression coefficient	Standard error	Chi-square value	Degrees of freedom	P value	OR	95% CI for OR		
							Lower	Upper	
Age	-0.02	0.02	0.49	1	p=0.49	0.98	0.94	1.03	
Diagnosis year			3.24	5	p=0.66				
Diagnosis year (1)	-0.66	0.54	1.50	1	p=0.22	0.52	0.18	1.48	
Diagnosis year (2)	-0.19	0.47	0.16	1	p=0.69	0.83	0.33	2.09	
Diagnosis year (3)	-0.21	0.51	0.16	1	p=0.69	0.82	0.30	2.20	
Diagnosis year (4)	-0.51	0.45	1.26	1	p=0.261	0.60	0.25	1.46	
Diagnosis year (5)	-0.63	0.46	1.85	1	p=0.174	0.53	0.22	1.32	
Race			0.91	2	p=0.633				
Race (1)	0.49	0.58	0.71	1	p=0.398	1.63	0.52	5.08	
Race (2)	0.76	1.47	0.26	1	p=0.608	2.13	0.12	37.85	
Income (1)	0.36	0.33	1.20	1	p=0.273	1.43	0.75	2.72	
Home location (1)	0.36	0.34	1.12	1	p=0.290	1.43	0.74	2.76	
GS (1)	-0.04	0.60	0.01	1	p=0.945	0.96	0.29	3.12	
PSA (1)	-1.27	0.44	8.24	1	p<0.01	0.28	0.12	0.67	
Constant	1.94	1.78	1.19	1	p=0.275	6.95			
									

Abbreviations: GS= Gleason Score; PSA= Prostate-specific antigen; CI=confidence interval; OR=odds ratio; PSM= propensity score matching.

**Table 4:** Multivariable Cox proportional hazard model for CSM and OM for CDSP (n=6,339 before PSM, and n=3177 after PSM) for unselected patients*.*

Outcomes	CDSP HR (95% CI)	P-value
CSM		
Non-adjusted	0.48 (0.43- 0.53)	p<0.001
Adjusted model 1	0.83 (0.73- 0.94)	p<0.05
Adjusted model 2	0.83 (0.73- 0.94)	p<0.05
PSM Non-adjusted	0.24 (0.19-0.31)	p<0.001
PSM Adjusted model 1	0.21 (0.16-0.27)	p<0.001
PSM Adjusted model 2	0.21 (0.16-0.27)	p<0.001
OM		
Non-adjusted	0.78 (0.73- 0.83)	p<0.001
Adjusted model 1	1.07 (0.99- 1.16)	p=0.08
Adjusted model 2	1.07 (0.99- 1.16)	p=0.09
PSM Non-adjusted	0.87 (0.79-0.95)	p<0.05
PSM Adjusted model 1	0.78 (0.71-0.85)	p<0.001
PSM Adjusted model 2	0.74 (0.73-0.81)	p<0.001

Adjusted model 1 adjusts for age, AJCC stage, GS, and race. Adjusted model 2 adjusts for age, AJCC stage, GS, race, diagnosis year, income, and home location. PSM-non-adjusted model adjusts for none. PSM-adjusted model 1 adjusts for age, AJCC stage, GS, and race. PSM-adjusted model 2 adjusts for age, AJCC stage, GS, race, diagnosis year, income, and home location. Abbreviations: HR=hazard ratio; PSM=propensity score matching (by1:1 matching); CDSP= Cancer-directed surgery performed; GS= Gleason Score; PSA= Prostate-specific antigen; AJCC stage =AJCC Stage 7th edition; CI=confidence interval; CSM=cancer-specific survival; OM=overall survival.

**Table 5:** Multivariable Cox proportional hazard model for CSM and OM for CDSP (n=185 before PSM, and n=177 after PSM) for young patients (≤74 years old) with AJCC stage IV diseases*.*

Outcomes	CDSP HR (95% CI)	P-value
CSM		
Non-adjusted	0.64 (0.51- 0.79)	p<0.001
Adjusted model 1	0.67 (0.53- 0.84)	p<0.001
Adjusted model 2	0.66 (0.52- 0.84)	p<0.001
PSM Non-adjusted	0.71 (0.53-0.95)	p<0.05
PSM Adjusted model 1	0.71 (0.53-0.95)	p<0.05
PSM Adjusted model 2	0.72 (0.53-0.97)	p<0.05
OM		
Non-adjusted	0.64 (0.52- 0.79)	p<0.001
Adjusted model 1	0.67 (0.54- 0.83)	p<0.001
Adjusted model 2	0.66 (0.53- 0.82)	p<0.001
PSM Non-adjusted	0.69 (0.53-0.90)	p<0.05
PSM Adjusted model 1	0.68 (0.52-0.90)	p<0.05
PSM Adjusted model 2	0.69 (0.53-0.91)	p<0.01

Adjusted model 1 adjusts for age, AJCC stage, GS, and race. Adjusted model 2 adjusts for age, AJCC stage, GS, race, diagnosis year, income, and home location. PSM-non-adjusted model adjusts for none. PSM-adjusted model 1 adjusts for age, AJCC stage, GS, and race. PSM-adjusted model 2 adjusts for age, AJCC stage, GS, race, diagnosis year, income, and home location. Abbreviations: HR=hazard ratio; PSM=propensity score matching (by1:1 matching); CDSP= Cancer-directed surgery performed; GS= Gleason Score; PSA= Prostate-specific antigen; AJCC stage =AJCC Stage 7th edition; CI=confidence interval; CSM=cancer-specific survival; OM=overall survival.
